# Isolation of a Stable Subpopulation of Mobilized Dental Pulp Stem Cells (MDPSCs) with High Proliferation, Migration, and Regeneration Potential Is Independent of Age

**DOI:** 10.1371/journal.pone.0098553

**Published:** 2014-05-28

**Authors:** Hiroshi Horibe, Masashi Murakami, Koichiro Iohara, Yuki Hayashi, Norio Takeuchi, Yoshifumi Takei, Kenichi Kurita, Misako Nakashima

**Affiliations:** 1 Department of Dental Regenerative Medicine, Center of Advanced Medicine for Dental and Oral Diseases, National Center for Geriatrics and Gerontology, Research Institute, Morioka, Obu, Aichi, Japan; 2 Department of Oral and Maxillofacial Surgery, School of Dentistry, Aichi-gakuin University, Nagoya, Aichi, Japan; 3 Department of Pediatric Dentistry, School of Dentistry, Aichi-gakuin University, Nagoya, Aichi, Japan; 4 Department of Endodontics, School of Dentistry, Aichi-g akuin University, Nagoya, Aichi, Japan; 5 Department of Biochemistry and Division of Disease Models, Center for Neurological Diseases and Cancer, Nagoya University Graduate School of Medicine, Nagoya, Aichi, Japan; Instituto Butantan, Brazil

## Abstract

Insights into the understanding of the influence of the age of MSCs on their cellular responses and regenerative potential are critical for stem cell therapy in the clinic. We have isolated dental pulp stem cells (DPSCs) subsets based on their migratory response to granulocyte-colony stimulating factor (G-CSF) (MDPSCs) from young and aged donors. The aged MDPSCs were efficiently enriched in stem cells, expressing high levels of trophic factors with high proliferation, migration and anti-apoptotic effects compared to young MDPSCs. In contrast, significant differences in those properties were detected between aged and young colony-derived DPSCs. Unlike DPSCs, MDPSCs showed a small age-dependent increase in senescence-associated β-galactosidase (SA-β-gal) production and senescence markers including *p16*, *p21*, *Interleukin (IL)-1β*, *-6*, *-8*, and *Groα* in long-term culture. There was no difference between aged and young MDPSCs in telomerase activity. The regenerative potential of aged MDPSCs was similar to that of young MDPSCs in an ischemic hindlimb model and an ectopic tooth root model. These results demonstrated that the stem cell properties and the high regenerative potential of MDPSCs are independent of age, demonstrating an immense utility for clinical applications by autologous cell transplantation in dental pulp regeneration and ischemic diseases.

## Introduction

Tissue regeneration and maintenance is dependent on mesenchymal stem cells (MSCs) [1]. For the application of MSCs in tissue engineering and regenerative medicine, it is important to optimize their isolation and preserve their phenotypic properties. Moreover, it is necessary to determine the influence of donor age on MSCs [2]. Aging related changes consist of three distinct types: quantity (proliferation potential), quality (differentiation/regenerative potential) and mobilization potential [3]. More information about the age-related changes in MSCs is essential for the successful development of cell-therapies for the aged [4].

In a recent study on human bone marrow MSCs (BMSCs) and adipose tissue derived MSCs (ASCs), it has been shown that MSC numbers decline with donor age [5], [6]. Aside from a decrease in overall expansion potential [7], several groups have also documented that aged MSCs have a decreased proliferation rate compared to young MSCs from the initial cell passages and cellular senescence [6], [8], [9]. In addition, telomere length is decreased in aged MSCs compared to young MSCs [9], and this telomere shortening has been shown to inhibit the mobilization of stem cells out of their niche [10]. Furthermore, the migratory activity of periodontal ligament stem cells (PDLSCs) is decreased with age [11] and gene expression of pro-angiogenic factors including VEGF, PlGF, and HGF in MSCs is down-regulated with increasing age [9], [12].

Results obtained from three different species -mouse, rat and human- clearly demonstrate a declining differentiation capability of MSCs with age [8], [11], [13], [14]. On the other hand, no changes were observed in MSCs with age has been reported in their osteogenic and adipogenic differentiation potential *in vivo* [15], [16] and *in vitro* [17], [18]. Furthermore, isolated CD105^+^ MSCs from the aged does not influence the adipogenic, myogenic [19], and osteogenic potential [20]. Thus, there appears to be contradictory results in the literature regarding the phenotypic properties of aged stem cells. These apparent discrepancies may be attributed to variable methods of isolation of MSCs, resulting in the coexistence of various subsets of stem cells that vary in their differentiation potential and their vulnerability to senescence. In addition, there is not yet a clear understanding of the different cellular markers of MSCs [19], [21].

Human dental pulp stem cells (DPSCs) represent a novel adult stem cell population that possesses the properties of high proliferative potential, self-renewal and multi-lineage differentiation [22]. The supply of autologous pulp tissue, however, is very limited with age, due to narrow root canals and the decrease in pulp tissue volumes due to physiological secondary dentin formation, pathological tertiary dentin formation and mineralization [23]. The older patients' teeth supply fewer colonies of human DPSCs than the younger patients' [24]. Human DPSCs from aged donors lose their proliferative activities and differentiation capabilities after repeated passages [25], [26]. Hypoxic cultures, grown under 3% O2, however, have succeeded in overcoming this deficiency, indicating the possibility to obtain optimal numbers of human DPSCs from aged patients [24]. We have devised a method of isolation of mobilized dental pulp stem cells (MDPSCs) by G-CSF-induced mobilization that generates stem cell enriched subpopulations with increased expression of angiogenic/neurotrophic factors, with higher trophic effect on migration, immunomodulation and anti-apoptosis and attendant angiogenic, neurogenic and regenerative potential [27]. Thus, the evidence in the literature that isolated subfractions of MSCs from the aged showed no change in differentiation potential compared with that from the young [19], [20] prompted us to determine whether the aged MDPSCs retain the quantity, quality and mobilization potential as the young MDPSCs useful for cell therapy.

Many factors are known to induce cellular senescence. These include dysfunctional telomeres, non-telomeric DNA damage, excessive mitogenic signals including those produced by oncogenes, perturbations to chromatin organization and stresses with an as-yet unknown etiology [28]. Generally, senescent cells display a characteristic enlarged, flattened morphology and are characterized by an irreversible G1 growth arrest involving the repression of genes that drive cell cycle progression and the up-regulation of cell cycle inhibitors like p53/p21 and p16/RB and by expression of senescence-associated β-galactosidase (SA-β-gal) activity [3], [29]. However, the expression of CXCR2 and its ligands such as IL-8, GROα is also up-regulated in senescence cells [30].

In the present investigation, we have compared the biological properties of aged MDPSCs versus young MDPSCs, including their phenotypic stability and the expression of senescence markers in long-term cultures. In addition the regenerative potential was examined in ischemic hindlimb and ectopic tooth root models. The results demonstrated that MDPSCs are efficiently enriched and stable, secreting high levels of trophic factors with high proliferation, migration, and regenerative potential, and that these properties of the MDPSCs are independent of age.

## Materials and Methods

This study was approved by the ethics committees and the animal care and use committees of National Center for Geriatrics and Gerontology, Research Institute and Aichi-gakuin University. All experiments were conducted using the strict guidelines of DNA Safety Programs.

### Isolation of DPSCs by G-CSF induced chemotaxis

Normal human third molars were collected from young adult patients (19–30 years of age, *n* = 6) and older patients (44–70 years of age, *n* = 6) at the Aichi-gakuin University Dental Hospital under approved guidelines set by School of Dentistry, Aichi-gakuin University and National Center for Geriatrics and Gerontology, Research Institute. Informed written consents were obtained from every donor for use of dental pulp tissue. Each dental pulp tissue were extracted from young and aged teeth and minced into pieces followed by enzymatic digestion using in 0.04 mg/ml Liberase (Roche, Mannheim, Germany) with slight modification of our previous method [31]. The number of isolated cells and their viability was determined by trypan blue staining of cells, and plated at 2–4×10^4^ cells on 35 mm dishes (Asahi Technoglass, Funabashi, Japan) in Dulbecco's Modified Eagle's Medium (DMEM) (Sigma, St. Louis, MO) supplemented with 10% human serum collected from healthy consenting adult donors and basic fibroblast growth factor (bFGF) (Fibrast splay, Kaken Pharmaceutiical Co. Ltd.) (10 ng/ml). The dental pulp stem cells forming colonies (DPSCs) were detached by incubation with TrypLE Select (Invitrogen) prior to 70% confluency and subcultured at 1–2×10^5^ cells on 10 cm dishes (Asahi Technoglass) in DMEM supplemented with 10% human serum.

The mobilized subpopulation of dental pulp stem cells, MDPSCs, was further isolated by our recently devised method utilizing G-CSF-induced stem cell mobilization [27]. Briefly, Costar Transwell (permeable support 8.0 µm polycarbonate membrane 6.5 mm Insert, Corning, Lowell, MA) as upper chamber was inserted into 24 well plate tissue culture plates (Falcon) as lower chamber. The membrane was modified chemically (Toray Industries, Inc., Tokyo) to prevent cell attachment. Young and aged DPSCs (2×10^4^ cells/100 µl DMEM) at the third passage of culture were seeded into the upper chambers respectively, and 390 µl DMEM supplemented with 10% human serum and G-CSF (100 ng/ml) was added in the lower chambers. After 48 h incubation, the medium was changed into DMEM supplemented with 10% human serum without G-CSF, and the numbers of the cells attached to the dishes were counted. After 7 days culture, aggregates of ≥50 cells were scored as colonies. Once cells reached 60–70% confluence, they were detached and subcultured until senescence. To evaluate the colony forming efficiency of MDPSCs, 1×10^3^ cells/ml of MDPSCs at the 4th passage were seeded into 35 mm dishes in DMEM supplemented with 10% human serum, and then incubated. After 4 days culture, aggregates of ≥10 cells were scored as colonies.

### Flow cytometric analysis

Both young and aged MDPSCs were characterized by a flow cytometry FACSAria II (BD Biosciences, San Jose, CA) at the 5th passage of culture as described previously [27], and young and aged DPSCs from the same individuals at the same passage were also used as controls. They were immunolabeled for 60 min at 4°C with antibodies against CD29, CD31, CD44, CD73, CD90, CD105, CD146, CXCR4, and G-CSFR, respectively.

### Induced differentiation

The differentiation of MDPSCs into angiogenic, neurogenic, odontogenic/osteogenic, and adipogenic lineages was induced and compared with that of DPSCs without the G-CSF induced migratory response [32].

### Real-time RT-PCR

Total RNA was extracted using Trizol (Invitrogen) from young and aged MDPSCs, and from young and aged DPSCs at the 6th passage. First-strand cDNA synthesis was performed on total RNA of those cells by reverse transcription using the ReverTra Ace-α (Toyobo, Tokyo, Japan) after DNase I treatment (Roche Diagnostics, Pleasanton, CA) at 37°C for 20 min. Real-time RT-PCR was performed as described previously [27] using the stem cell markers, *Oct3/4*, *Nanog*, *Sox2*, *Rex1*, *Stat3*, and *CXCR4*. To examine mRNA expression of angiogenic and neurotrophic factors, real-time RT-PCR amplifications of *granulocyte-monocyte colony-stimulating factor* (*GM-CSF*), *matrix metalloproteinase* (*MMP*)*-3*, *vascular endothelial growth factor* (*VEGF*), *brain-derived neurotrophic factor* (*BDNF*), *glial cell derived neurotrophic factor* (*GDNF*), *nerve growth factor* (*NGF*), and *neurotrophin-3* (*NT-3*) were also performed [27].

### Proliferation and migration assay

To determine the proliferative activity in response to G-CSF, aged MDPSCs were compared with young MDPSCs at the 5th passage at 3×10^3^ cells per 96 well in DMEM without serum. Young and aged DPSCs from the same individuals were used as controls. Ten µl of Tetra-color one (Seikagaku Kogyo, Co., Ltd) were added to the 96 well plate, and cell numbers were measured using a spectrophotometer at 450 nm absorbance at 2, 12, 24, 36, and 48 h of culture. Wells without cells served as negative controls.

To examine the migratory activity of young and aged MDPSCs, horizontal chemotaxis assay using TAXIScan-FL (Effector Cell Institute) was performed as described previously [33]. Young and aged DPSCs from the same individuals were also used as controls.

### Effect of MDPSCs-conditioned medium

At 60% confluence, the culture medium was switched to DMEM without serum and the conditioned media from young and aged MDPSCs, and young and aged DPSCs at the 5th to 6th passage were collected 24 h later, and concentrated about 40 times by Amicon Ultra-15 Centrifugal Filter Unit with Ultracel-3 membrane (Millipore, Billerica, MA).

To assess the immunomodulatory effect of the conditioned medium (CM) of MDPSCs, a mixed lymphocyte reaction (MLR) assay was performed as described previously [34]. Autologous PBMCs and allogenic stimulator PBMCs were co-cultured at 10^4^ cells per 96 well in RPMI-1640 without arginine, leucine, lysine, and phenol red (Sigma-Aldrich) supplemented 5 µg/ml of each CM. Cell numbers were measured at 0, 12, 24, and 36 h.

To assess the anti-apoptotic effect of the CM, NIH3T3 cells were incubated with 500 nM staurosporine (Sigma-Aldrich) in DMEM supplemented with 5 µg/ml of CM from young and aged MDPSCs and from young and aged DPSCs. After 3 h, NIH3T3 cells were harvested, and the cell suspensions were treated with Annexin V-FITC (Roche Diagnostics) and propidium iodide for 15 min, and analyzed by flow cytometry.

### Stability of MDPSCs phenotype

To evaluate the stability of aged MDPSCs, the 6th passage of MDPSCs were compared with the 12th passage in the proliferation and migratory activities. Flow cytometric analysis of cell surface markers (CD29, CD31, CD44, CD73, CD90 and CD105) was also performed in young and aged MDPSCs to compare the 6th passage with the 12th passage.

Senescence associated (SA)-β-gal (Senescence Cells Histochemical Staining Kit, Sigma-Aldrich, St. Louis, MO, USA) staining assay and real-time RT-PCR analysis of senescence related genes (*p16, p21, Interleukin-1β* (*IL-1β*), *Interleukin-6* (*IL-6*), *Interleukin-8* (*IL-8*) and *Growth related oncogene-α* (*GROα*) were performed in the 6th and 20th passages of young MDPSCs and DPSCs in comparison with aged MDPSCs and DPSCs as described previously [27].

To analyze the karyotype of aged MDPSCs at the 7th passage, chromosomes prepared from cells were stained with quinacrine mustard (Sigma-Aldrich) and Hoechst 33258 (Sigma-Aldrich) dissolved in a McIlvane's buffered solution (pH 7.0) and examined under a fluorescence microscope. Suitable metaphases were photographed and karyotypes were analyzed.

The telomerase activity was determined with a Quantitative Telomerase detection kit (Allied Biotech, Inc., Vallejo, CA) as described previously [27]. Briefly, the 8th, 12th and 20th passages of MDPSCs and DPSCs from young and aged donors were lysed in Lysis buffer and the telomerase activity was determined with a Quantitative Telomerase detection kit (Allied Biotech, Inc., Vallejo, CA) by Applied Biosystems 7500 Real-time PCR system (Applied Biosystems, Foster City, CA) using whole-cell extract containing 0.5 µg of protein.

The telomere length was analyzed using the Telo TAGGG telomere Length Assay kit according to manufacturer's instructions (Roche Applied Science, Indianapolis, IN). Genomic DNA (gDNA) was extracted from the 8th, 12th and 20th passages of MDPSCs and DPSCs from young and aged donors using a DNA extraction kit (Life Technologies Corporation, Carlsbad, CA USA). One microgram of gDNA was digested with the mixture of HinfI and RsaI restriction endonucleases, electrophoresed through a 0.8% agarose gel, and transferred to a nylon membrane to be hybridized to a digoxigenin (DIG)-labeled telomeric oligonucleotide (TTAGGG)_3_. The DNA/oligonucleotide hybridization products were visualized after reaction with a chemiluminescent substrate, using ImageQuant LAS4000 (GE Healthcare Life Sciences, Little Chalfont, UK).

### Transplantation of MDPSCs into a mouse ischemic hindlimb model

The angiogenic potential of MDPSCs was examined in a murine model of hind limb ischemia in 5-week-old severe combined immunodeficient (SCID) mice (CB17, CLEA, Tokyo) as described previously [32]. In brief, 24 h after ligation of the left proximal portion of the femoral artery, 1×10^6^ cells of young and aged MDPSCs, and young and aged DPSCs at the 6th passage after DiI (Sigma) labeling were injected intramuscularly. PBS injection was also used as negative control. Laser Doppler imaging (Perimed AB, Stockholm, Sweden) was performed 14 days after cell transplantation. The cryosections (12 µm thick) of isolated muscle tissue of ischemic hind limb were prepared and immunostained with Fluorescein *Griffonia (Bandeiraea) simplicifolia* Lectin 1/fluorescein-*Galanthus nivalis* (snowdrop) lectin (20 mg/ml; Vector Laboratories, Inc., Youngstown, OH). Microscopic digital images of six sections of every 120 µm were scanned in a frame composed of 500 µmx380 µm rectangle and statistical analyses was performed using software Dynamic cell count, BZ-HIC, on a fluorescein microscope BIOREVO, BZ-9000 (KEYENCE, Osaka, Japan). The co-localization of DiI-labeled transplanted cells and the newly formed BS-1 lectin-positive capillaries were observed using confocal laser microscopy (TCS SP5 conventional inverted microscope, Leica Microsystems, Wetzlar, Germany).

### Subcutaneous implantation of human tooth root in SCID mice

An experimental model of human tooth root was used for the evaluation of ectopic pulp regeneration after subcutaneous implantation into SCID mice as described previously [27]. Human tooth roots were prepared, 6 mm in length, 1 mm in width, and one end was sealed with cement. Both young and aged MDPSCs, and young and aged DPSCs, respectively, at the 7 th passage, 1×10^6^ cells each, were injected into the root with collagen TE (Nitta Gelatin), and each 4 roots were transplanted subcutaneously into 5-week-old SCID mice (CB17, CLEA). The four roots in which only collagen TE was injected were also transplanted as a control. Sixteen roots were harvested for histology after 21 days. The paraffin sections (5 µm in thickness) were stained with hematoxylin and eosin (HE), and the relative amounts of regenerated pulp tissue were morphometrically analyzed in each sample. For vascular staining, 5-µm-thick paraffin sections were immunostained with RECA1 (MONOSAN, UDEN, Netherlands) (1∶500) and 2nd antibody (Vector) (1∶200) and examined by confocal laser microscopy (TCS SP5 conventional inverted microscope, Leica). The ratio of RECA1 positive newly formed capillaries to the root canal area in the aged MDPSCs transplantation was compared with that in the young MDPSCs transplantation and the young DPSCs transplantation using a fluorescence microscope (BIOREVO, BZ-9000, KEYENCE).

For the analysis of regenerated dentin, *in situ* hybridization was performed using an odontoblastic marker, enamelysin. DIG signals were detected using a TSA system. Regenerated dentin width and enamelysin-positive cells along dentinal wall in the regenerated area on day 28 were calculated in three sections of each tooth (*n* = 4 teeth) by LAS AF software (Leica) using confocal laser microscopy.

### Statistical Analyses

Data are reported as mean ± SD. *P* values were calculated using Student's *t* test and Tukey's multiple comparison test method in SPSS 21.0 (IBM, Armonk, NY http://www.ibm.com). The number of replicates in each experiment is indicated in the figure legends.

## Results

### Morphological analysis of young and aged MDPSCs

The efficiencies of attachment of young and aged mobilized DPSCs through membrane with G-CSF (MDPSCs) were 5% and 3%, respectively. Aged MDPSCs were morphologically similar to young MDPSCs, containing stellate cells with long process and spindle-shaped cells (**Figure S1**). Limiting dilution analysis at the third passage culture showed that the frequencies of colony forming unit (CFU) in young and aged MDPSCs were estimated to be 88% and 87%, respectively, and those in young and aged DPSCs were 70% and 68%, respectively.

### Flow cytometric analysis of young and aged MDPSCs

Evaluation of the “stemness” of MDPSCs from aged donors was performed by flow cytometric analysis in comparison with that of young MDPSCs, and young and aged DPSCs. Young and aged MDPSCs and DPSCs were positive for CD29, CD44, CD73 and CD90, and negative for CD31, which are minimal criteria for MSCs. It is noteworthy, however, that the positive rates of CD105, CXCR4 and G-CSFR of young and aged MDPSCs were similar and were significantly higher than those of young and aged DPSCs. On the other hand, the positive rates of CD105 and G-CSFR of aged DPSCs were significantly lower compared to those of young DPSCs. These results suggested that aged MDPSCs contained similar numbers of pulp stem/progenitor cell populations to young MDPSCs, and that stemness may depend on the isolation method (**Table 1**).

**Table 1:** Flow cytometric analysis of cell surface markers on MDPSCs compared with DPSCs isolated from young and aged donors.

	Young (n = 6)				Aged (n = 6)			
	MDPSCs		DPSCs		MDPSCs		DPSCs	
	Positive (%)	SD	Positive (%)	SD	Positive (%)	SD	Positive (%)	SD
CD29	97.8%	1.4	98.0%	1.5	97.3%	1.2	97.5%	2.1
CD31	0.1%	0.1	0.2%	0.2	0.2%	0.2	0.2%	0.2
CD44	98.8%	2.3	98.8%	2.2	99.5%	0.7	99.6%	0.5
CD73	98.0%	1.5	98.9%	1.0	97.5%	2.8	97.6%	2.5
CD90	98.0%	2.4	98.7%	1.4	97.0%	3.7	97.6%	3.0
CD105	##96.6%	2.2	**64.7%	7.6	##91.9%	2.9	48.6%	6.9
CD146	17.1%	8.1	16.4%	4.2	8.9%	3.7	21.4%	6.8
CXCR4	##16.5%	2.9	7.3%	1.8	##16.1%	2.0	7.6%	1.1
G-CSFR	^#^56.7%	11.3	*26.0%	8.1	##44.2%	9.7	11.9%	3.7

## *P*<0.01, MDPSCs versus DPSCs;

**P<0.01,

*P<0.05, young DPSCs versus aged DPSCs.

### Multi-lineage differential potential of aged MDPSCs

**Figure 1:**
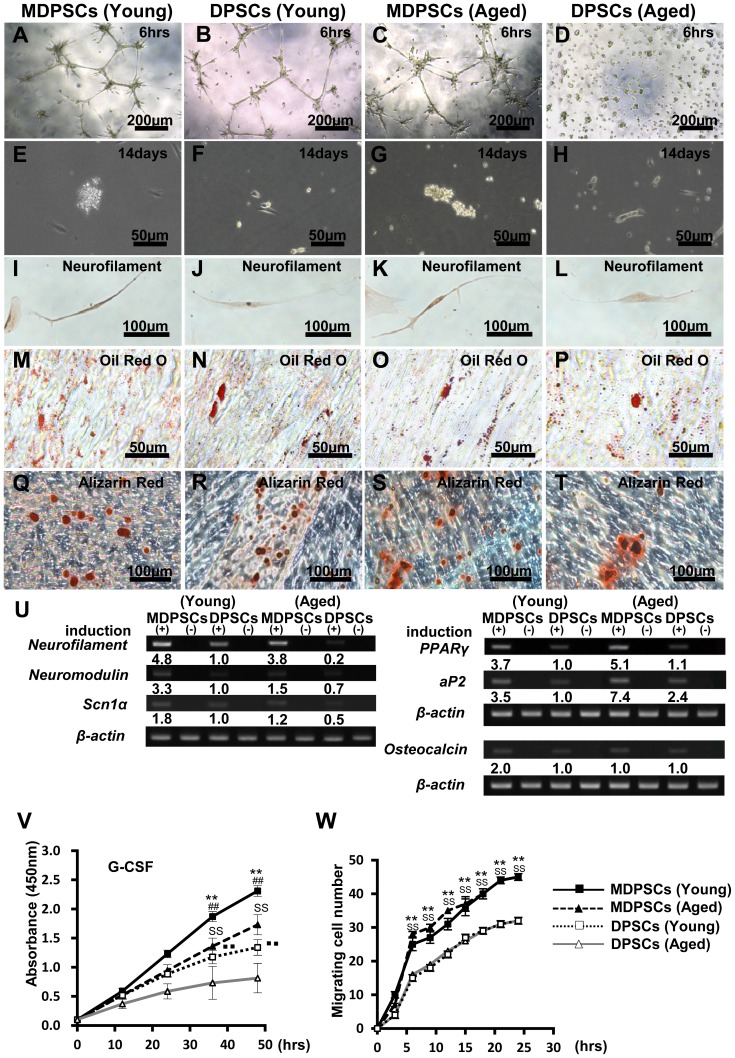
Multi-lineage differentiation potential and the characteristics. The aged dental pulp stem cells (DPSCs) mobilized by granulocyte-colony stimulating factor (G-CSF) (MDPSCs) were compared with aged colony-derived DPSCs (DPSCs), young MDPSCs and young DPSCs. (A–D) The endothelial differentiation potential using the matrigel assay. (E–H) Neurosphere formation, 14 days after induction. (I–L) Neuronal differentiation potential. Fourteen days after induction of dissociated neurosphere cells. (M–P) Adipogenic differentiation potential. (Q–T) Odontoblast differentiation potential. (U) Gene expression of *neurofilament*, *neuromodulin*, and *sodium channel, voltage-gated type I α* (*SCN1A*) for neuronal markers, *peroxisome proliferator-activated receptor γ* (*PPARγ*) and *adipocyte fatty acid binding protein 2* (*aP2*) for adipogenic markers, *osteocalcin* for a odonto/osteoblastic marker. (V) The proliferation analysis stimulated by 10% human serum (**p<0.01, young MDPSCs versus young DPSCs; ^SS^ *p*<0.01, aged MDPSCs versus aged DPSCs; ^##^ *p*<0.01, young MDPSCs versus aged MDPSCs; ^▪▪^ *p*<0.01, young DPSCs versus aged DPSCs). (W) The migration analysis stimulated by G-CSF (10 ng/ml) (**p<0.01, young MDPSCs versus young DPSCs; ^SS^ *p*<0.01, aged MDPSCs versus aged DPSCs). Data are expressed as the means ± SD of 6 determinations. The experiments were repeated six times (6 lots), and one representative experiment is presented.

The multi-lineage differential potential of young and aged MDPSCs was compared to that of young and aged DPSCs. Aged MDPSCs formed extensive networks of cords and tube-like structures on a matrigel as early as 6 h like young MDPSCs (**Figure 1A, C**). However, no such formation was detected in aged DPSCs (**Figure 1D**). Fourteen days after neurogenic induction, clusters of proliferating neurospheres were more prevalent in aged MDPSCs and in young MDPSCs compared with DPSCs both from aged and young donors (**Figure 1E–H**). Following neuronal induction, neurite outgrowth and positive immunostaining for neurofilaments were found in aged MDPSCs as those in young MDPSCs (**Figure 1I, K**). The mRNA expression of the neuronal markers *neurofilament*, *neuromodulin*, and *sodium channel, voltage-gated type 1α* (*Scn1A*) in aged MDPSCs was similar to that of young MDPSCs and was higher compared with that of aged and young DPSCs (**Figure 1U**). As for the adipogenic induction, there was little difference among MDPSCs and DPSCs both from aged and young donors, as shown by the positive staining by Oil red O (**Figure 1M–P**) and the expression of the adipogenic markers *PPARγ* and *aP2* mRNA (**Figure 1U**). Aged MDPSCs, however, had the highest expression of these adipogenic markers (**Figure 1U**). Finally, 28 days after osteogenic/dentinogenic induction, the mineralized matrix was stained by alizarin red (**Figure 1Q–T**), expressing the osteoblastic marker, *osteocalcin* mRNA, similarly in all cell populations (**Figure 1U**).

### Biological characteristics of young and aged MDPSCs

The proliferation activity with human serum was higher in MDPSCs than in DPSCs from both young and aged donors. There were, however, significant differences between aged and young MDPSCs (*P*<0.05) (**Figure 1V**). The migratory activity with G-CSF was much higher in MDPSCs than in DPSCs and there was no significant difference between young and aged MDPSCs (**Figure 1W**).

**Figure 2:**
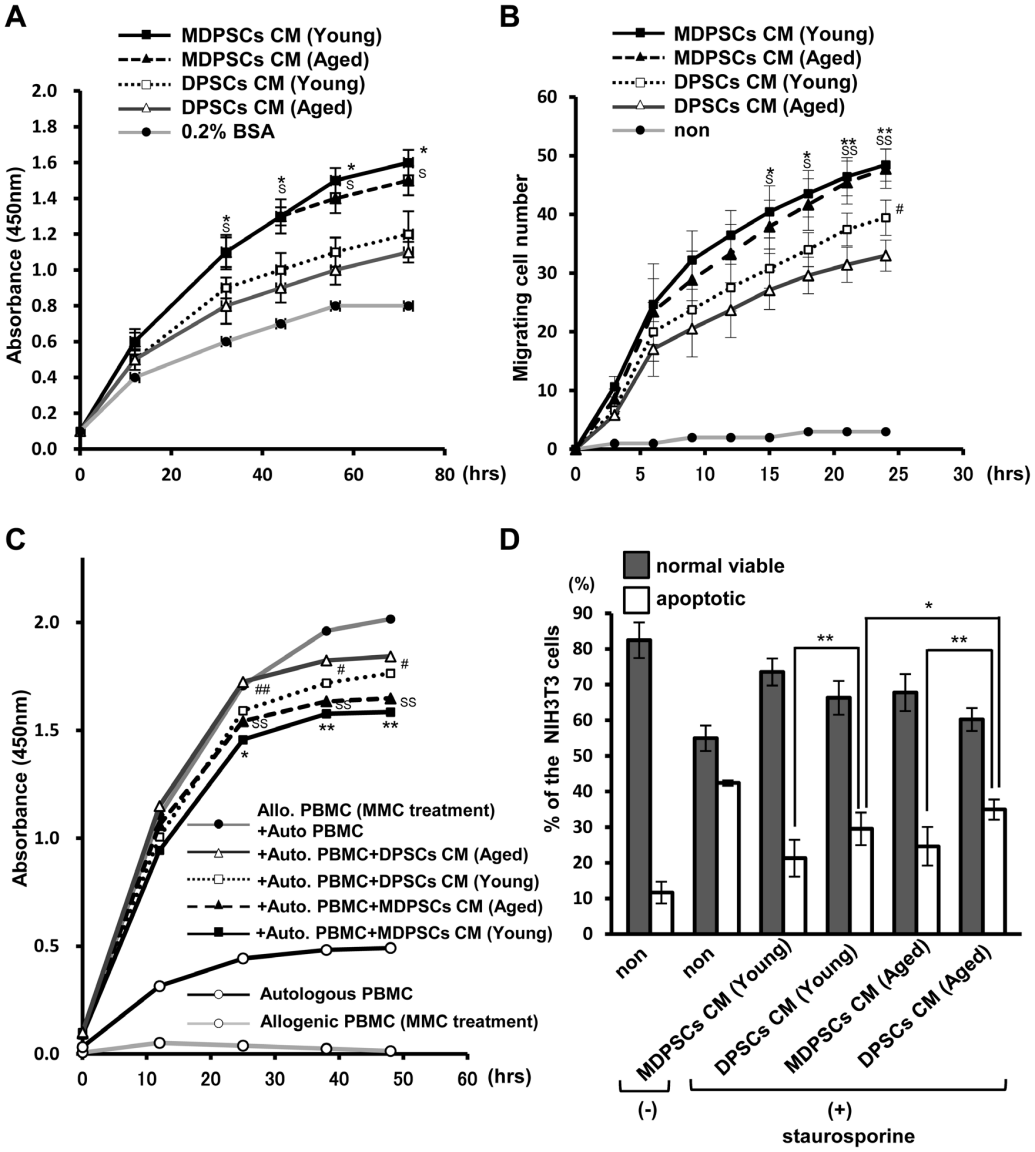
Effect of conditioned medium (CM) of aged MDPSCs. Effect of conditioned medium (CM) of the aged MDPSCs compared with those of aged DPSCs, young MDPSCs and young DPSCs. (A) The proliferative effect in NIH3T3 cells. (*p<0.05, young MDPSCs CM versus young DPSCs CM; ^S^ *p*<0.05, aged MDPSCs CM versus aged DPSCs CM). (B) The migratory effect in NIH3T3 cells (*p<0.05, **p<0.01, young MDPSCs CM versus young DPSCs CM; ^S^ *p*<0.05, ^SS^ *p*<0.01, aged MDPSCs CM versus aged DPSCs CM; ^#^ *p*<0.05, young DPSCs CM versus aged DPSCs CM). (C) Mixed lymphocyte reaction (MLR) analysis. (*p<0.05, **p<0.01, young MDPSCs CM versus young DPSCs CM; ^SS^ *p*<0.01, aged MDPSCs CM versus aged DPSCs CM; ^#^ *p*<0.05, ^##^ *p*<0.01, young DPSCs CM versus aged DPSCs CM). (D) The relative percentage of viable and apoptotic cells analyzed by flow cytometry by Annexin V staining. *p<0.05, **p<0.01. Data are expressed as the means ± SD of 6 determinations. The experiments were repeated three times (6 lots), and one representative experiment is presented.

Next, the ability of the conditioned medium (CM) from aged MDPSCs to accelerate cell proliferation and migration and suppress immunoreaction and apoptosis was investigated. The CM of aged MDPSCs was significantly more effective on proliferation compared to the CM of aged DPSCs. There was no significant difference in proliferation between the CM from aged and young MDPSCs (**Figure 2A**). The migratory effect of the CM from aged MDPSCs was higher than that from aged DPSCs when assayed on NIH3T3 cells. It is noteworthy that there was no significant difference between young and aged MDPSCs (**Figure 2B**). A significantly enhanced immunosuppression by the CM of MDPSCs was shown by MLR assay compared to the CM of DPSCs. No significant difference in immunosuppression between young and aged MDPSCs was found (**Figure 2C**). The survival rate of NIH3T3 cells was significantly enhanced by the CM of MDPSCs compared to the CM of DPSCs both from aged and young donors. The anti-apoptotic effect of the CM of aged MDPSCs was similar to that of the CM of young MDPSCs (**Figure 2D**). These results suggest that aged MDPSCs may induce similar trophic effects as young MDPSCs.

The mRNA expression of the stem cell markers, *Oct3/4*, *Nanog*, *Sox2*, *Rex1*, *Stat3* and *CXCR4* was similar in aged MDPSCs to those in young MDPSCs (Table 2), suggesting a similar stemness for aged MDPSCs and young MDPSCs. The expression of the angiogenic and/or neurotrophic factors *GM-CSF*, *MMP3*, *VEGF*, *BDNF*, *GDNF*, *NGF* and *NT-3* was similar or a little higher in young MDPSCs compared to aged MDPSCs (Table 2), suggesting that MDPSCs may have similar angiogenic/vasculogenic and neurogenic potential. On the other hand, the expression of the stem cell markers, *Sox2* and *CXCR4*, and the angiogenic/neurotrophic factors, *GM-CSF*, *MMP3*, *VEGF*, *BDNF*, *GDNF* and *NT-3* was much higher in aged MDPSCs than those in aged DPSCs. These results suggest a marked increase in the functionality and quality of MDPSCs compared with DPSCs from the aged donors.

**Table 2:** Relative mRNA expression of stem cell markers and angiogenic/neurotrophic factors in young MDPSCs, aged MDPSCs and aged DPSCs compared with young DPSCs.

Genes	Young		Aged	
	MDPSCs	DPSCs	MDPSCs	DPSCs
*Oct3/4*	2.6	1.0	1.2	0.6
*Nanog*	1.4	1.0	1.0	0.4
*Sox2*	6.7	1.0	7.9	0.5
*Rex1*	2.0	1.0	1.5	0.8
*Stat3*	2.8	1.0	1.6	0.6
*CXCR4*	14.4	1.0	5.6	0.1
*GM-CSF*	6.0	1.0	4.3	0.7
*MMP3*	25.5	1.0	24.9	0.8
*VEGF*	2.2	1.0	2.0	0.6
*BDNF*	2.3	1.0	1.1	0.7
*GDNF*	1.8	1.0	0.9	0.6
*NGF*	2.0	1.0	2.5	0.9
*NT-3*	1.3	1.0	1.4	0.4

The experiments were repeated three times (6 lots), and one representative experiment is presented.

### Maintenance of the MDPSCs characteristics

**Figure 3:**
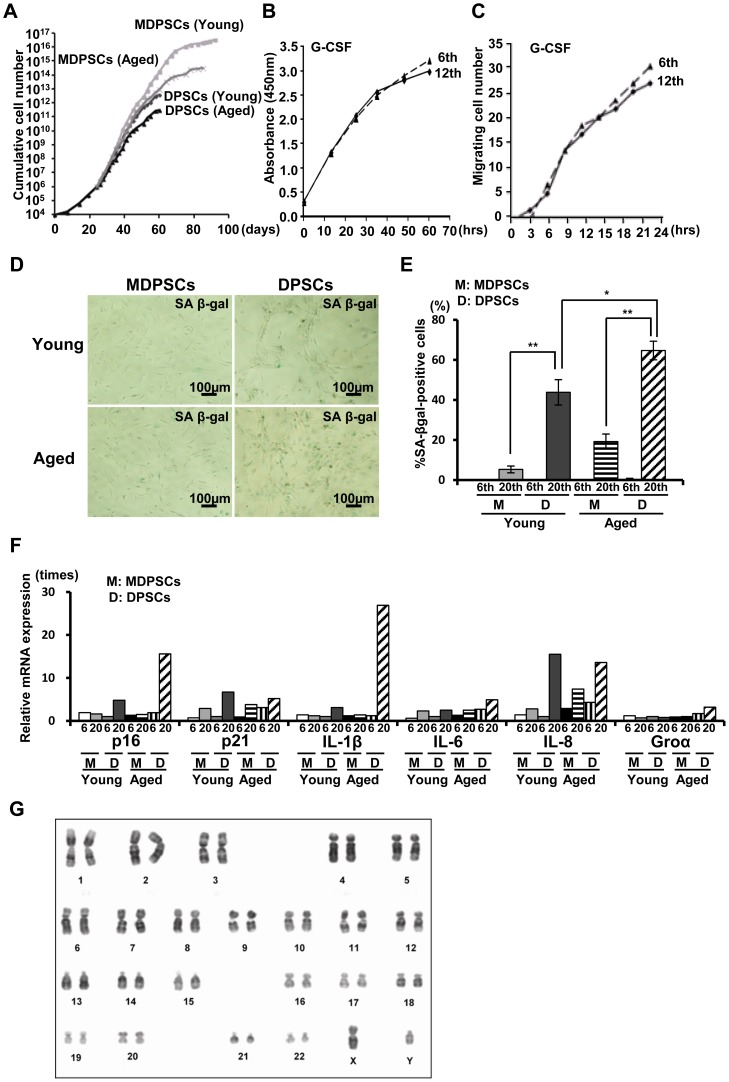
Maintenance of the stem cell properties of the aged MDPSCs with prolonged culture. (A) The cumulative cell number of aged MDPSCs, aged DPSCs, young MDPSCs and young DPSCs. (B) The proliferation of aged MDPSCs at the 6th and 12th passages by 10% human serum. (C) Migration of aged MDPSCs at the 6th and 12th passages. (D) Senescent associated (SA)-β-gal staining. (E) Percentage of SA-β-gal-positive cells of aged and young MDPSCs, and aged and young DPSCs at the 20th passage. *p<0.05, **p<0.01. Data are expressed as the means ± SD of 6 determinations. The experiments were repeated three times (6 lots), and one representative experiment is presented. (F) Relative mRNA expression of senescence-related genes, *p16*, *p21*, *IL-1β*, *IL-6*, *IL-8* and *Groα* in aged and young MDPSCs, and aged and young DPSCs at the 6th and 20th passages. The experiments were repeated six times (6 lots), and one representative experiment is presented. (G) Q-banding analysis for aged MDPSCs at the 7th passage showing normal karyotype.

The cumulative cell number of MDPSCs was much higher, and the proliferative life span of MDPSCs was longer than those of DPSCs both from aged and young donors. Aged MDPSCs showed a lower cumulative cell number compared to young MDPSCs (**Figure 3A**). To examine the stability of aged MDPSCs after prolonged *ex-vivo* culture, the proliferation activity, migratory activity, and cell surface markers in the 6th passage of aged MDPSCs were compared with those in the 12th passage. There was no significant difference between 6th and 12th passage of aged MDPSCs in proliferation activity (**Figure 3B**), migratory activity (**Figure 3C**) and percentages of cell surface markers (**Table S1**). The number of senescence associated (SA)-β-gal positive cells increased in DPSCs at the 20th passage, but both aged and young MDPSCs were negative for SA-β-gal expression at the 20th passage (**Figure 3D, E**). Furthermore, the expression of *p16*, *p21*, *IL-1β*, *IL-6*, *IL-8* and *Groα* was higher in aged DPSCs compared with that in aged MDPSCs at the 20th passage (**Figure 3F**). There were also no chromosomal abnormalities/aberrations in the karyotype of aged MDPSCs at the 7th passage (**Figure 3G**). Taken together, these results indicate stability of MDPSCs after cell expansion irrespective of age.

**Figure 4:**
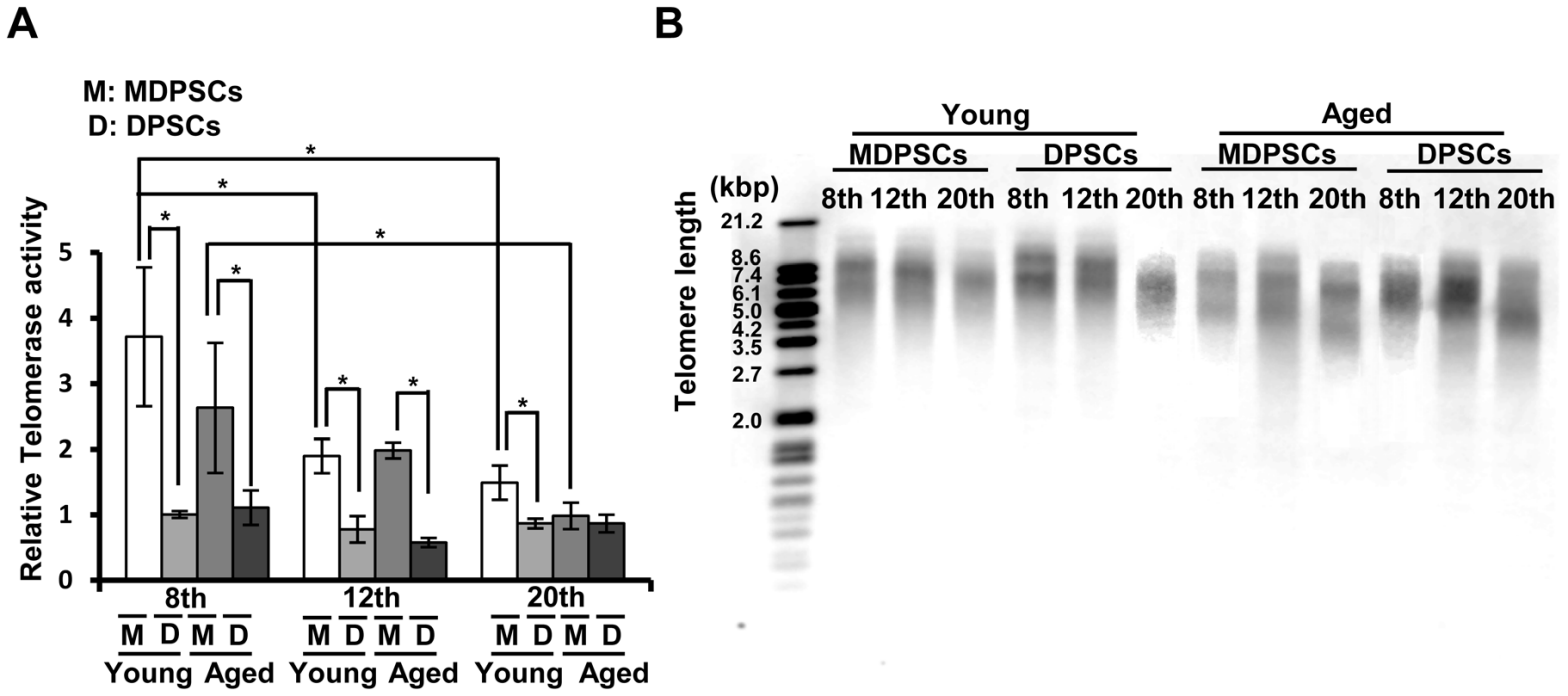
Changes of the telomerase activity and the telomere length with prolonged culture. (A) Relative telomerase activity in aged and young MDPSCs, and aged and young DPSCs at the 8th, 12th and 20th passages. *p<0.05. Data are expressed as the means ± SD of 3 determinations. The experiments were repeated four times (4 lots), and one representative experiment is presented. (B) Southern blot analysis of telomeres in aged and young MDPSCs, and aged and young DPSCs at the 8th, 12th and 20th passages. Molecular sizes (kbp) are indicated on the left. The experiments were repeated four times (4 lots), and one representative experiment is presented.

The telomerase enzyme activity of young and aged MDPSCs at the 8th, 12th and 20th passages was analyzed in comparison with that of young and aged DPSCs at each passage. Our results showed a reduction in both young and aged DPSCs compared to that in young and aged MDPSCs. In addition, the telomerase activity gradually decreased in MDPSCs with increasing passage number (**Figure 4A**). The telomere length was monitored using the mean terminal restriction fragment (TRF) length of genomic DNA isolated from young and aged MDPSCs at the 8th, 12th and 20th passages in comparison with that of young and aged DPSCs at each passage. The telomere length was longer both in young and aged MDPSCs compared to that in young and aged DPSCs. With prolonged culture, the telomere length gradually decreased in MDPSCs (**Figure 4B**), consistent with the result of the telomerase activity.

### Neovascularization in ischemic hindlimb with MDPSCs transplantation

**Figure 5:**
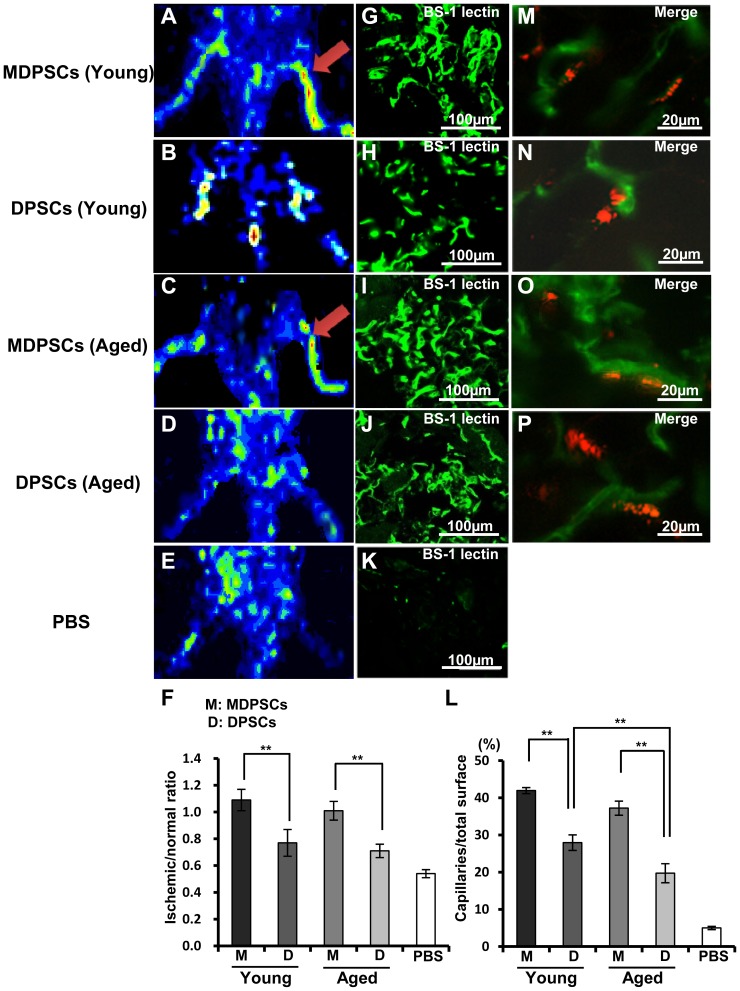
Neovascularization in the ischemic hindlimb 14 days after transplantation of aged MDPSCs. Neovascularization in the ischemic hindlimb 14 days after transplantation of aged MDPSCs compared with those of aged DPSCs, young MDPSCs and young DPSCs. (A–E) Laser Doppler imaging. Accelerated blood flow (arrows). (F) Quantification of blood flow in the ischemic versus normal limbs obtained from four mice in each group. **p<0.01. (G–K) Immunostaining of Fluorescein Griffonia (Bandeiraea) Simplicifolia Lectin 1/fluorescein-galanthus nivalis (snowdrop) lectin (BS-1 lectin) in the ischemic hindlimb. (L) Quantification and statistical analysis of the capillary density in the ischemic region using serial sections. Data are expressed as means ± SD of 4 determinations. **p<0.01. The experiments were repeated three times (3 lots), and one representative experiment is presented. (M–P) Localization of DiI-labeled transplanted cells and newly formed capillaries stained by BS-1 lectin.

Next, the regenerative potential of human MDPSCs was determined in a murine model of hindlimb ischemia. Fourteen days after transplantation of young and aged MDPSCs, the quantitative analysis of blood flow and capillary density was performed in comparison with young and aged DPSCs. Laser Doppler imaging revealed that blood flow was significantly increased approximately 1.4 and 2.0 times more in aged MDPSCs transplantation compared with aged DPSCs and PBS control without cells, respectively (**Figure 5C, D, E, F**). Capillary density in the ischemic region transplanted with aged MDPSCs increased 2.1 times higher than that with aged DPSCs (**Figure 5I, J, L**). There was no significant difference in blood flow and capillary density between young and aged MDPSCs (**Figure 5F, 5L**). Confocal microscopic analysis showed that DiI-labeled MDPSCs and DPSCs both from aged and young donors did not co-localize with BS-1 lectin stained blood vessels (**Figure 5M–P**), implying their trophic effect on neovascularization.

### Ectopic pulp regeneration by aged MDPSCs in the tooth root

**Figure 6:**
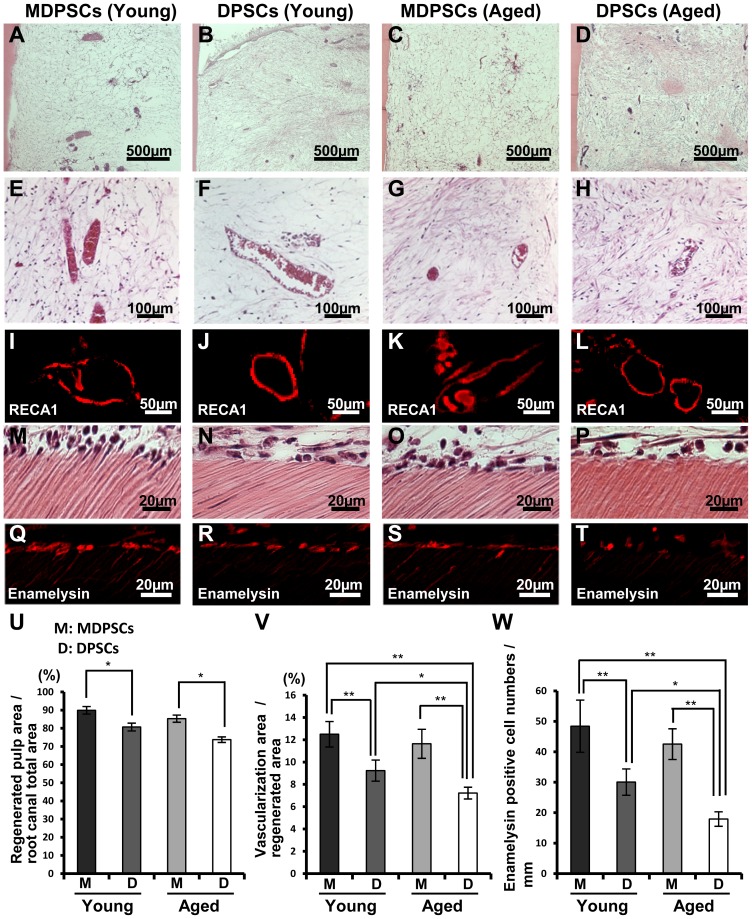
Regeneration of pulp tissue after ectopic tooth transplantation in severe combined immunodeficiency (SCID) mice. Aged MDPSCs, aged DPSCs, young MDPSCs and young DPSCs were injected into the emptied root canals. (A–L) Hematoxylin and Eosin (HE) staining. (I–L) Immunostaining with RECA1. (M–P) Odontoblast-like cells lining to the dentinal wall. (Q–T) In situ hybridization analysis with *enamelysin*. (U) Ratio of the regenerated area to the root canal area. Data are expressed as means ± SD of four determinations. *p<0.05. (V) Ratio of the vascularization area to the regenerated area. Data are expressed as means ± SD of four determinations. *p<0.05, **p<0.01. (W) *Enamelysin* positive cell number of the dentinal wall. Data are expressed as means ± SD of four determinations. *p<0.05, **p<0.01.

We next evaluated pulp regenerative potential of human MDPSCs in an experimental model of ectopic tooth root transplantation in SCID mice. Pulp-like tissue with well-organized vasculature was regenerated in the tooth root 28 days after transplantation of MDPSCs and DPSCs from aged donors similar to young donors (**Figure 6A–H**). Statistical analysis showed that the regenerated pulp area was significantly larger (1.2-fold) in the transplantation of aged MDPSCs compared to the transplantations of aged DPSCs, and was similar to the comparison of young MDPSCs and DPSCs (**Figure 6U**). Neovascularization was observed in the regenerated pulp tissue after all the cell transplantations by immunofluorescent staining analysis with RECA1 antibody (**Figure 6I–L**). Statistical analysis showed that the vascularization areas were significantly larger (1.5-fold) in the transplantations of aged MDPSCs compared to aged DPSCs, and was similar to the comparison of young MDPSCs and young DPSCs (**Figure 6V**). Odontoblastic differentiation along the dentinal wall was also detected in the transplantations of aged MDPSCs with values similar to those of young MDPSCs and DPSCs, and was higher than that of aged DPSCs (**Figure 6M–T, W**), demonstrating higher dentin regeneration potential of aged MDPSCs compared with aged DPSCs.

## Discussion

The aim of this investigation was to determine the influence of age on the mobilized dental pulp stem cells (MDPSCs). It is well known that there is a decline in MSCs properties [35]. In the present investigation, however, we have demonstrated that MDPSCs from aged donors are similar to young donors in their capacities for migration, differentiation, expression of angiogenic/neurotrophic factors, trophic effects on proliferation and migration, and anti-apoptotic effects. MDPSCs from aged and young donors expressed stem cell markers, CD105, CXCR4 and G-CSFR. On the other hand, age-related changes of these capacities were demonstrated in colony-derived non- mobilized DPSCs. Similar age-independency in stemness has been reported in isolated MSCs [19], [20]. Thus, isolation using G-CSF mobilization renders the DPSCs to overcome the decline in stemness occurring with age. The present study demonstrated that the isolation utilizing G-CSF-induced stem cell mobilization [27] result in enrichment of G-CSFR positive DPSC subsets similarly both from young and aged donors. G-CSF promotes cell proliferation, migration, anti-apoptosis, endothelial cell differentiation and immunomodulation [27]. Thus, the present result that there was no significant difference in those stem cell properties between young MDPSCs and aged MDPSCs is plausible.

Tissue regeneration is dependent on stem cells and therefore, any loss in number or functionality due to aging will likely have a profound effect on regenerative capacity [36]. Aging of MSCs accompanied by a decline in the regenerative potential has been reported in many studies in vivo [11], [24], [37]. Therefore, in the present study, the regenerative potential for pulp/dentin and stem cell functionality of MDPSCs from the aged were examined in an ectopic tooth transplantation model compared with the young. It is noteworthy that no significant differences were found between them. On the other hand, a significant decline in pulp regenerative potential of DPSCs from the aged donors was demonstrated compared to that from the young donors. Thus, the induction of the migratory response by G-CSF in the DPSCs (MDPSCs) nullified the observed age dependent differences in the DPSCs. The angiogenic/vasculogenic potential of the aged MDPSCs transplanted in an ischemic hindlimb model was also similar to that of the young MDPSCs, while there was a significant difference between the aged and the young DPSCs. MSCs possess trophic activities including enhanced proliferation [38], [39], enhanced migration [39], anti-inflammatory [40] and anti-apoptotic effects [41], and similar trophic activities have also been reported for MDPSCs [27]. In addition, the present study has shown that conditioned medium of aged MDPSCs had similar trophic effects *in vitro* as those from the young, whereas conditioned medium of aged DPSCs showed inferior trophic effects than those of the young. Furthermore, it has been shown that the regenerative potentials are dependent on trophic effects of MSCs [42]. Thus, the findings in this study suggest that donor age has no effect on the regeneration potential of MDPSCs and in their trophic effects.

Constitutive expression of telomerase RT (TERT) subunit gene prevents senescence and is responsible for the maintenance of MSCs properties. Telomere length, their shortening rate and activity of telomerase can be useful markers for aging evaluation [2], [8]. Similarly, it has been well documented that the production of senescence-associated β-galactosidase, which is caused by related increased lysosomal activities and altered cytosolic pH, increases with aging [16], [43]. In the present study, little accumulation of SA-β-gal was detected in aged MDPSCs even at the 20th passage, as in young MDPSCs, whereas an increased rate in aged DPSCs was observed. Gradual up-regulation of p16 and p21, which is associated with growth arrest [44], and pro-inflammatory cytokines IL-6 and IL-8, was also demonstrated in aged DPSCs but not in aged MDPSCs during cell expansion. These findings are consistent with a higher mRNA expression of telomerase RT (TERT) in aged MDPSCs compared with aged DPSCs, which prevents senescence and is responsible for the maintenance of stem cell properties. Telomeres are shortened by approximately 17 bps every year over a lifetime and make MSCs more sensitive to apoptotic stimuli [45]. There are, however, conflicting reports on the effect of aging on telomere length in MSCs [19], [46]. The present study has demonstrated that telomere length was slightly shorter in aged MDPSCs at the 20th passage of culture compared with those at the 8th passage. These findings suggest that isolated MDPSCs might be superior to colony-derived DPSCs in inhibiting senescence and in maintaining functional characteristics of stemness during aging.

Finally, the proportion of people over age 60 is growing rapidly in industrial countries including Japan, and innovative strategies of stem cell therapy for the aged are a high priority. During aging, the status of MSCs changes with respect to their self-renewal capability, migration and differentiation potential, and the production of trophic factors that characterize their microenvironment [47]. Age-related modification of MSCs properties should be considered for suitable autologous cell transplantation. The method for isolation of DPSCs subsets on their migratory response to G-CSF is safe, efficacious and reproducible as described previously [27]. It is noteworthy, that in this investigation we have established unequivocally that the stem cell properties and their regenerative potential are significantly superior in aged MDPSCs compared to aged DPSCs, demonstrating their utility for potential clinical applications to autologous cell transplantation, especially in aged patients.

## Conclusions

Comparison of human MDPSCs from aged donors to young donors demonstrated that there was a little age-related decline in stem cell properties including migration and differentiation potential, and in trophic effects on proliferation, migration, and anti-apoptosis. MDPSCs also showed a small age-dependent increase in senescence-associated β-galactosidase (SA-β-gal) production and senescence markers in long-term culture. Furthermore, the regenerative potential of aged MDPSCs was also similar to that of young MDPSCs in an ischemic hindlimb model and an ectopic tooth root model. Thus, these results demonstrated minimal alteration in human MDPSCs with aging, suggesting an immense utility for MDPSCs in clinical applications in dental pulp regeneration and ischemic diseases by autologous cell transplantation.

## Supporting Information

Figure S1 **Young and aged MDPSCs at the 3rd passage of culture forming a colony on day 3.**
(TIF)

Table S1 **Flow cytometric analysis of cell surface markers on young and aged MDPSCs at the 12 th passage compared with those at the 6 th passage.** The experiments were repeated three times (3 lots), and one representative experiment is presented.
(TIF)
