# Correction: Isolation of a Stable Subpopulation of Mobilized Dental Pulp Stem Cells (MDPSCs) with High Proliferation, Migration, and Regeneration Potential Is Independent of Age

**DOI:** 10.1371/journal.pone.0151741

**Published:** 2016-03-11

**Authors:** Hiroshi Horibe, Masashi Murakami, Koichiro Iohara, Yuki Hayashi, Norio Takeuchi, Yoshifumi Takei, Kenichi Kurita, Misako Nakashima

The authors would like to correct panel B of Fig 4, as errors were introduced during the preparation of this figure for publication. Please view the corrected Fig 4 here. The authors confirm that this change does not alter the conclusions of the paper.

**Fig 4:**
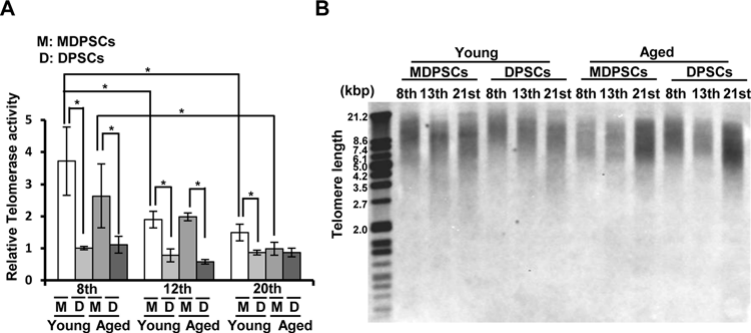
Changes of the telomerase activity and the telomere length with prolonged culture. (A) Relative telomerase activity in aged and young MDPSCs, and aged and young DPSCs at the 8th, 12th and 20th passages. *p<0.05. Data are expressed as the means ± SD of 3 determinations. The experiments were repeated four times (4 lots), and one representative experiment is presented. (B) Southern blot analysis of telomeres in aged and young MDPSCs, and aged and young DPSCs at the 8th, 12th and 20th passages. Molecular sizes (kbp) are indicated on the left. The experiments were repeated four times (4 lots), and one representative experiment is presented.
