# Supplementary figures and images for: Isolation of a Stable Subpopulation of Mobilized Dental Pulp Stem Cells (MDPSCs) with High Proliferation, Migration, and Regeneration Potential Is Independent of Age
Source: PLoS One. 2014 May 28;9(5):e98553. doi: 10.1371/journal.pone.0098553 (PMC4037225; doi:10.1371/journal.pone.0098553)

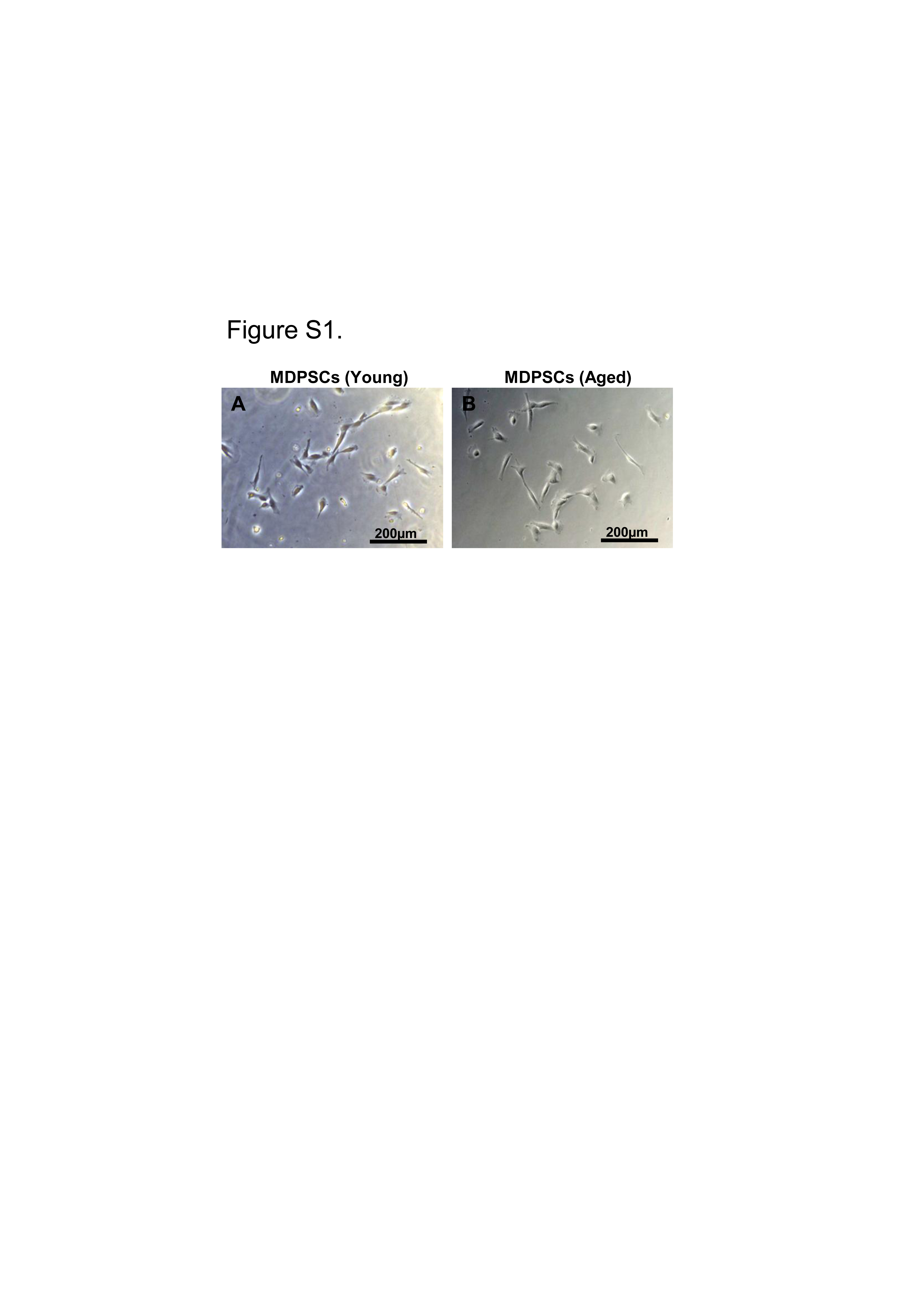

Supplement: Figure S1 — Young and aged MDPSCs at the 3rd passage of culture forming a colony on day 3. (TIF) [file pone.0098553.s001.tif]

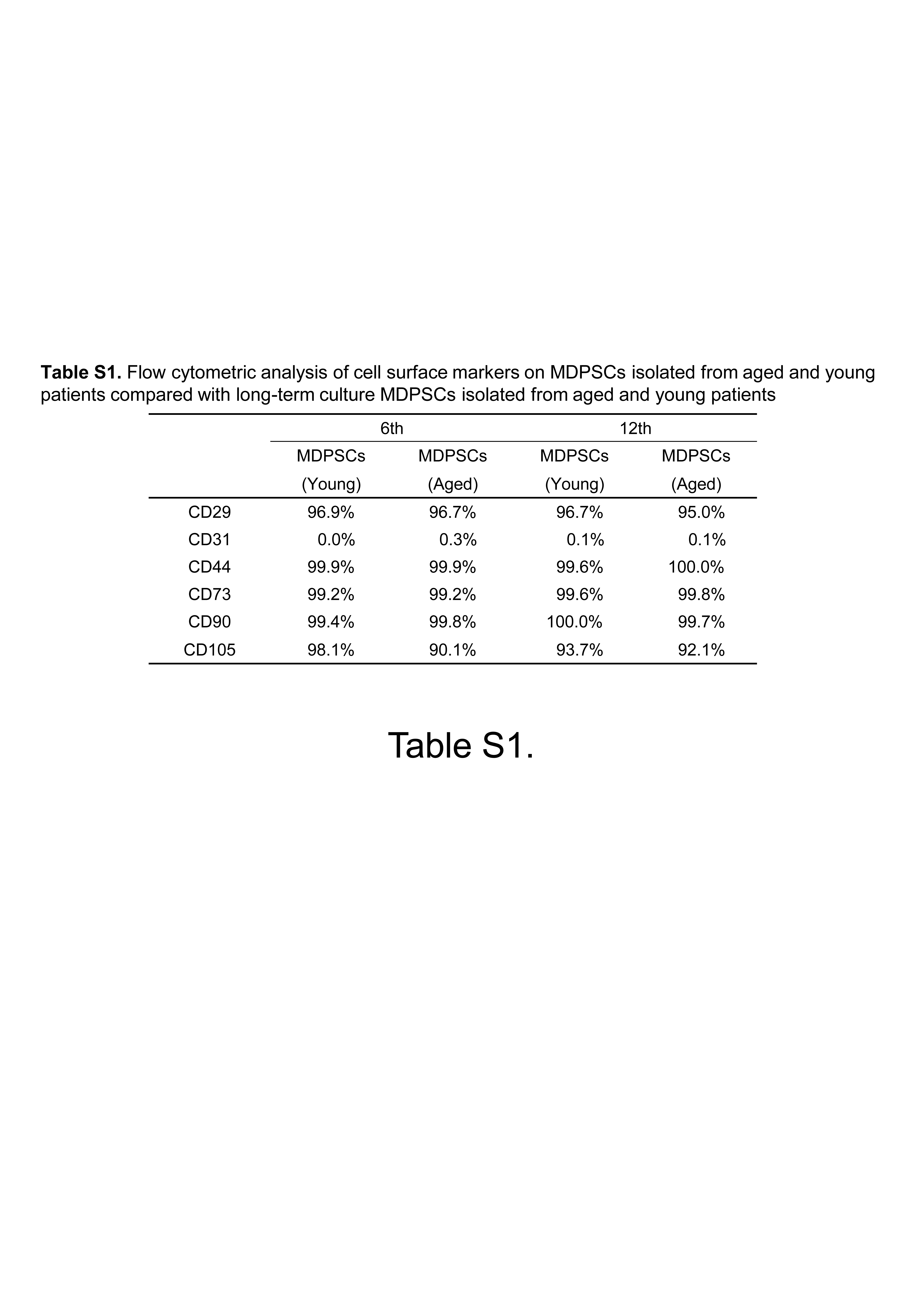

Supplement: Table S1 — Flow cytometric analysis of cell surface markers on young and aged MDPSCs at the 12 th passage compared with those at the 6 th passage. The experiments were repeated three times (3 lots), and one representative experiment is presented. (TIF) [file pone.0098553.s002.tif]
